# CD4^+^ and CD8^+^ T cells have opposing roles in breast cancer progression and outcome

**DOI:** 10.18632/oncotarget.3958

**Published:** 2015-04-29

**Authors:** Yi Huang, Chunling Ma, Qunyuan Zhang, Jian Ye, Fang Wang, Yanping Zhang, Pamela Hunborg, Mark A. Varvares, Daniel F. Hoft, Eddy C. Hsueh, Guangyong Peng

**Affiliations:** ^1^ Department of Internal Medicine, Saint Louis University School of Medicine, Saint Louis, MO, USA; ^2^ Center for Clinical Molecular Medicine, Children's Hospital of Chongqing Medical University, Chongqing, P. R. China; ^3^ Department of Laboratory Medicine, Women and Children's Health Care Hospital of Linyi City, Linyi, P. R. China; ^4^ Molecular Biology Experimental Center, Shandong Medical College, Linyi, P. R. China; ^5^ Department of Genetics, Washington University School of Medicine in St. Louis, Saint Louis, MO, USA; ^6^ Department of Laboratory Medicine, The First Affiliated Hospital of Nanjing Medical University, Nanjing, P. R. China; ^7^ Department of Surgery, Saint Louis University School of Medicine, Saint Louis, MO, USA; ^8^ Department of Otolaryngology-Head and Neck Surgery, Saint Louis University School of Medicine, Saint Louis, MO, USA

**Keywords:** CD4^+^ T cells, CD8^+^ T cells, regulatory T cells, Th17 cells, breast tumor microenvironment

## Abstract

The Cancer Immunoediting concept has provided critical insights suggesting dual functions of immune system during the cancer initiation and development. However, the dynamics and roles of CD4^+^ and CD8^+^ T cells in the pathogenesis of breast cancer remain unclear. Here we utilized two murine breast cancer models (4T1 and E0771) and demonstrated that both CD4^+^ and CD8^+^ T cells were increased and involved in immune responses, but with distinct dynamic trends in breast cancer development. In addition to cell number increases, CD4^+^ T cells changed their dominant subsets from Th1 in the early stages to Treg and Th17 cells in the late stages of the cancer progression. We also analyzed CD4^+^ and CD8^+^ T cell infiltration in primary breast cancer tissues from cancer patients. We observed that CD8^+^ T cells are the key effector cell population mediating effective anti-tumor immunity resulting in better clinical outcomes. In contrast, intra-tumoral CD4^+^ T cells have negative prognostic effects on breast cancer patient outcomes. These studies indicate that CD4^+^ and CD8^+^ T cells have opposing roles in breast cancer progression and outcomes, which provides new insights relevant for the development of effective cancer immunotherapeutic approaches.

## INTRODUCTION

Breast cancer is the second leading cause of cancer-related death in women worldwide. Significant progress has been made in the diagnosis and treatments of human breast cancer, but clinical outcomes of patients are still discouraging [1, 2]. Immunotherapy is a highly attractive alternative approach to treat patients with advanced breast cancer [3-9]. Increasing evidence suggests that interplay between immune cells and tumor cells exerts a major influence on breast tumor development and progression [10]. Furthermore, recent “Cancer Immunoediting” concept provided insights that immune system has both immune surveillance and tumor promotion effects during the cancer development [11-14]. Thus, a better understanding of the dynamic roles and pathogenesis of immune cells, especially T cells in breast cancer is essential for the development of novel strategies to treat cancer patients.

It is established that an effective anti-tumor immune response requires the involvement of both CD4^+^ and CD8^+^ T cells [11-14]. The role of CD4^+^ T cells in anti-tumor immunity has recently been extensively studied in both pre-clinial animal models and and clinical cancer patients. CD4^+^ T cells are critical for priming of tumor-specific CD8^+^ T cells and for the seconary expansion and memory of CD8^+^ T cells as well [15, 16]. However, the discovery of regulatory T cells (Treg) and Th17 cells not only changes the classical Th1/Th2 paradigm of Th cell differentiation, but also markedly alters conventional thinking regarding the role of CD4^+^ T cells in anti-tumor immunity [17-21]. It has been shown that tumor-infiltrating Treg cell-induced immunosuppressive microenvironment prevents effective anti-tumor immunity and becomes a major obstacle to the success of immunotherapy against breast cancer [21-25]. Furthermore, the functional contribution of human Th17 cells to tumor immunity remains controversial since both pro- and anti-tumor effects have been observed varied among tumor types [19, 20]. Therefore, improved understanding of the nature and functional roles of these CD4^+^ T cell subsets in tumor immunity during the course of breast cancer development is important for effective treatments of breast cancer. However, little is known about the dynamic and fuctional alterations of these T cell subsets in the immunoediting processes during breast cancer progression. In addition, very little information is available for the understanding of which T cell subset is the determinant for clinical outcomes of breast cancer.

Increasing evidence suggests that tumor-specific tumor-infiltrating T cells (TILs) but not circulating T cells predict clinical outcomes of cancer patients [26-28]. Studies from the retrospectively analyzed clinical tumor tissues of breast cancer patients further demonstrated that tumor-infiltrating CD8^+^ T cells and FoxP3^+^ T cells are important components for assessing disease prognosis and clinical progression [29-32]. However, the dynamic distribution and quantity of these T cell subsets in different organs and tumor tissues with the tumor progression have not been reported in breast cancer. Suitable animal models are required to precisely determine prognostic roles of CD4^+^ and CD8^+^ T cells with different distribution origins in breast cancer, which should not only provide new prognostic information but also be a key for the development and clinical application of novel immunotherapeutic strategies in human breast cancer.

To better understand the roles of CD4^+^ and CD8^+^ T cells in the pathogenesis of breast cancer, we utilized 4T1 and E0771, two distinct mouse breast cancer models to mimic different clinical stages of human breast cancer. We further characterized the dynamic distributions of CD4^+^ T cell subsets and CD8^+^ T cells in different organs with varied cancer developmental stages in these two mouse models. We showed that both CD4^+^ and CD8^+^ T cells involved the immune responses but with distinct dynamic trends in breast cancer development, suggesting their potentially different roles in directing breast cancer progression. In addition to cell number increases, CD4^+^ T cells changed their dominant subsets from Th1 in the early stages to Treg and Th17 cells in the late stages of the breast cancer development. Moreover, we determined CD4^+^ T cell subset and CD8^+^ T cell infiltration in primary breast cancer tissues from cancer patients and retrospectively analyzed their correlations with prognostic factors and clinical outcomes. Our results further confirmed that CD8^+^ T cells are the key effector cell population and have positive effects on anti-tumor immunity and patient clinical outcomes, and that CD4^+^ and CD8^+^ T cells have opposing prognostic effects for breast cancer patient outcomes. These studies collectively suggest the dynamics and opposing roles of CD4^+^ and CD8^+^ in directing breast cancer progression and outcomes.

## RESULTS

### Accumulation of both CD4^+^ T and CD8^+^ T cells but with different trends in the tumor microenvironment during breast cancer development and progression

**Figure 1:**
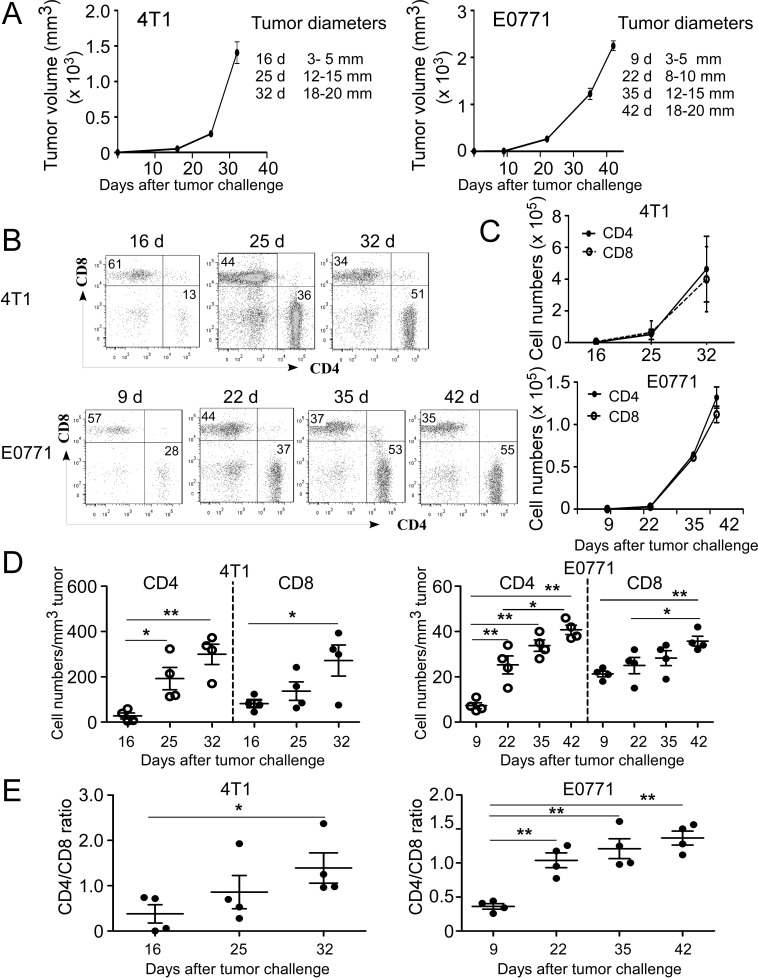
Accumulation of both CD4^+^ and CD8^+^ T cells in the TILs during tumor development and progression in breast cancer mouse models **A.** Tumor growth curves of mouse breast cancer 4T1 and E0771 cells were determined based on tumor sizes. 4T1 (1×10^5^ cells/mouse) and E0771 (2×10^5^ cells/mouse) were implanted into mammary gland fat pads of female BALB/c and C57BL/6 mice (*n* = 4), respectively. When primary tumors reached indicated sizes, tumor-bearing mice and tumor free-littermate controls were sacrificed, TILs were isolated and cell proportions and numbers analyzed. **B.** The proportions of CD4^+^ and CD8^+^ T cells in TILs were analyzed at the indicated time points using flow cytometry analyses by gating CD3^+^ population. Results shown are a representative graph of 4 tumor-bearing mice. **C.** Increased absolute cell numbers of both CD4^+^ and CD8^+^ TILs with the tumor progression. Tumor-infiltrating CD4^+^ and CD8^+^ T cells were calculated based on their proportions in CD3^+^ T cells and total cell numbers in each digested tumor tissue. **D.** Relative cell numbers of both CD4^+^ and CD8^+^ TILs with the tumor progression were calculated based on their absolute cell numbers per tumor volume in each tumor tissue. **E.** Dynamic changes of CD4^+^ to CD8^+^ T cell ratios at the different stages of tumor development. Result of each dot shown in **D.** and **E.** is derived from an individual mouse. Data shown are mean ± SE from four mice in each time point. *p < 0.05 and **p < 0.01 between the indicated two groups determined by paired student's t test. Data shown in **A.** to **E.** are representative from three independent experiments with similar results.

To better understand the interactions and role of immune system in the pathogenesis of breast cancer, two distinct murine mammary cancer cell lines 4T1 and E0771 with different pathologic types were selected to establish breast cancer models for our studies. 4T1 cells originally isolated from BALB/c mice is a good model for metastatic and advanced stages of breast cancer developing tumor with a rapid progression [33]. While E0771 cell is medullary breast adenocarcinoma cells from C57BL/6 mice, representing a good model for spontaneously developed breast cancer [34]. To mimic different tumor stages of cancer progression in the clinical cancer patients, varied tumor sizes instead of growth times were utilized for the time points in this study (Figure 1A). When tumor volumes reached the indicated sizes after tumor challenge, T cell numbers and proportions were analyzed in the different organs and tumor sites. Gradually increased cell proportions and absolute numbers of tumor-infiltrating CD4^+^ and CD8^+^ T cells were observed in both 4T1 and E0771 tumor-bearing mice with the increasing tumor size and progression, indicating accumulation of both CD4^+^ and CD8^+^ T cells into tumor sites (Figure 1B and 1C). To exclude the possibility that increased absolute T cell numbers are due to the enlarged tumors, the relative cell numbers per tumor volume of CD4^+^ and CD8^+^ TILs were further determined at different tumor stages. We observed consistent trends as shown in the absolute numbers that relative cell numbers of CD4^+^ and CD8^+^ TILs were also significantly increased in both 4T1 and E0771 mouse models during the course of tumor development (Figure 1D). However, we found that CD4^+^ and CD8^+^ TILs had distinctly different patterns during tumor progression, indicating different roles of these two cell populations in the pathogenesis of breast cancer (Figure 1B–1D). In the early stages of tumor development (before 16 days in 4T1 and 22 days in E0771 models), CD8^+^ T cells were more dominant than CD4^+^ T cells in TILs; while the reversed proportion was observed in the late stages of tumor development. Furthermore, more rapidly increased infiltration of CD4^+^ T cells than that of CD8^+^ T in the tumor sites was observed with the tumor progression (Figure 1D). These results were further confirmed in the analyses of the CD4/CD8 T cell ratios in the different tumor stages based on their proportions and absolute cell numbers in TILs. As expected, markedly increased CD4/CD8 ratios at the late tumor stages were observed compared with those in the early tumor stages in both 4T1 and E0771 breast tumor models (Figure 1E). Collectively, both CD4^+^ and CD8^+^ T cells were accumulated in the tumor microenvironment but with different increased trends during breast cancer development and progression.

We also determined whether the phenomena and alterations of CD4^+^ and CD8^+^ T cells observed in TILs were also applied to the T cells in the peripheral organs, including in peripheral blood, spleen and draining lymph nodes. We found similar trends of CD4^+^ and CD8^+^ T cells in bloods as those in TILs, showing significantly increased CD4/CD8 T cell ratios with the advanced tumor stages both in 4T1 and E0771 mouse models (Supplemental Figure 1A). However, the trends and phenomena were not observed in CD4^+^ and CD8^+^ T cells obtained from spleens and lymph nodes in both tumor models (Supplemental Figure 1B and 1C). These data suggested that varied changes and different roles of T cells may exist which depend on their origins and organ locations.

### Dynamics and distinct distributions of tumor-infiltrating CD4^+^ T cell subsets during breast cancer development and progression

**Figure 2:**
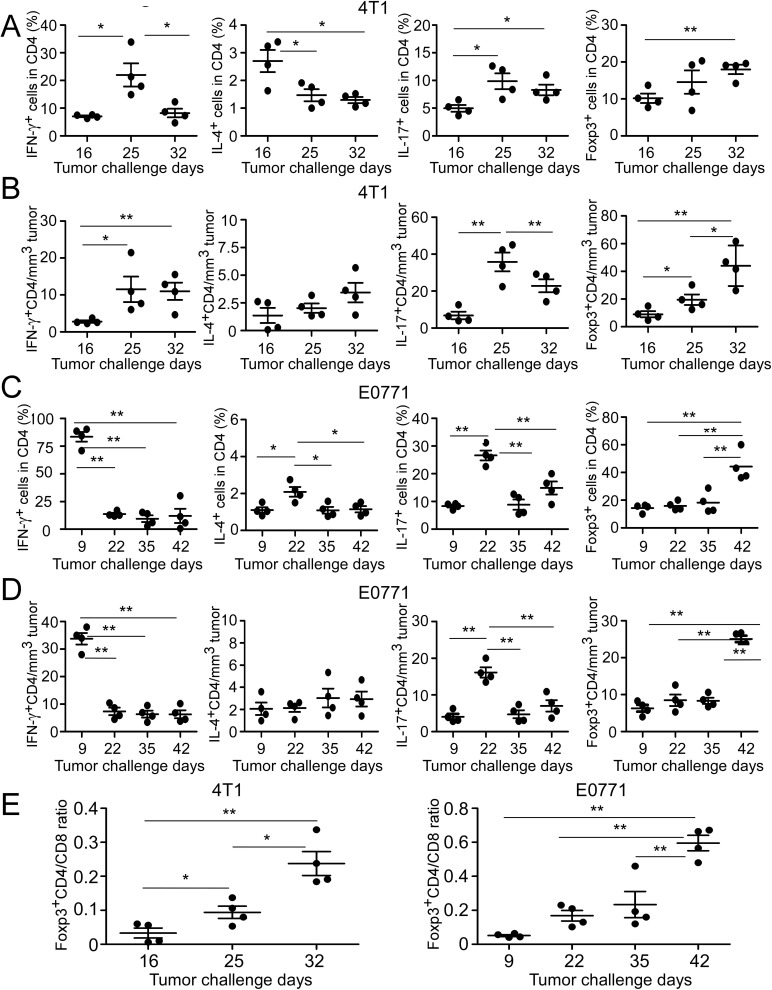
Dynamics of tumor-infiltrating CD4^+^ T cell subsets in mouse breast cancer models **A.** and **C.** Proportions of CD4^+^ T cell subsets in TILs were analyzed at the indicated time points using flow cytometry analyses by gating CD4^+^ population. The treatment procedure was identical as that described in Figure 1. TILs were isolated and intracellular staining performed after stimulation with PMA and ionomycin for 5 hours. **B.** and **D.** Relative cell numbers of CD4^+^ T cell subsets with the tumor progression were calculated based on their absolute cell numbers per tumor volume in each tumor tissue. **E.** Dynamic changes of Foxp3^+^CD4^+^ to CD8^+^ T cell ratios were analyzed based on the proportions of CD4^+^Foxp3^+^ subset and CD8^+^ TILs. Result of each dot shown in **A.** to **E.** is derived from an individual mouse. Data shown are mean ± SE from four mice in each time point. *p < 0.05 and **p < 0.01 between the indicated two groups determined by paired student's *t* test. Data shown in **A.** to **E.** are representative from three independent experiments with similar results.

Accumulating evidence suggest that CD4^+^ T cells play a critical role for tumor immunity and each subset has a unique role in adaptive immune during the tumor development [11–14]. Given that our results showed significantly increased CD4^+^ T cell proportion and numbers in TILs of late stages of breast cancer progression, we reasoned that CD4^+^ T cell subsets and their roles may alter during breast cancer progression, resulting in tumor promotion rather than tumor surveillance. To test this possibility, we evaluated the dynamic distributions of CD4^+^ T cell subsets based on their proportions and relative cell numbers per tumor size at different stages of cancer development in these two breast cancer models (Supplemental Figure 2A and 2B). We expectedly observed that tendency of CD4^+^IFN-γ^+^ T cells, both in fraction and relative cell numbers were significantly increased in the early and middle cancer stages. However, both then were declined with the advanced stages in the two breast tumor models, suggesting their important role as effector T cells involved in early tumor surveillance (Figure 2A–2D). Furthermore, in the E0771 model, the peak of CD4^+^IFN-γ^+^ was observed much earlier than that in the 4T1 tumor model with tumor progression, suggesting the dynamic differences of Th1 cells in these two models (Figure 2C and 2D). For IL-4^+^CD4^+^ subset, although it also showed decreased proportions, no significant difference was observed in its relative cell numbers in both 4T1-bearing and E0771-bearing mice (Figure 2A–2D). Notably, not only the proportion but also total cell numbers of IL-4^+^CD4^+^ cells remained in a relatively low level among TILs during the breast cancer development, suggesting that Th2 subset is a subdominant subset compared with the other T cell subsets ( < 4%) (Supplemental Figure 2C).

Th17 cells have been extensively studied in mouse tumor models and human cancer patients during the past several years, but conclusions regarding the functional role of Th17 cells in tumor immunity remain controversial [19]. Our previous studies have shown increased Th17 cells in human breast cancer TILs [35, 36]. We observed that the proportion and relative cell numbers of CD4^+^IL-17^+^ subset dramatically went up at the early stage and reached a peak at the middle cancer stage, and then significantly dropped down at the late stage of tumor progression in both 4T1 (Figure 2A and 2B) and E0771 tumor models (Figure 2C and 2D). CD4^+^IL-17^+^ T cell subset had a similar tend as that of CD4^+^IFN-γ^+^ subset in the absolute cell numbers following the tumor development (Supplemental Figure 2C), but its percentage and relative cell numbers maintained relative higher levels in the late cancer stage compared with IFN-γ^+^CD4^+^T cells (Figure 2A–2D). The results suggest that Th17 cells become a dominant CD4^+^ T cell population in TILs of breast cancer.

It has been clear that FoxP3^+^ Treg-induced immune suppression is the major obstacle for successful anti-tumor immunity [21–25]. In contrast to the other T cell subsets, FoxP3^+^CD4^+^ Treg subpopulation had a constantly higher proportion (10-40%) in CD4^+^ T cell subsets from initial stage to late stage with the tumor progression (Figure 2A and 2C). More strikingly, its proportion and relative cell numbers both showed significant increases following tumor progression and reached the highest level at the late cancer stage in both 4T1 and E0771 tumor models (Figure 2A–2D). In addition, absolute Foxp3^+^ Treg cell numbers revealed a rapid increase compared with that of the other T cell subsets in the tumor sites (Supplemental Figure 2C), further confirming that Treg cells are the most dominant CD4^+^ T cell subset forming a tumor suppressive microenvironment at late stage of tumor progression. Given that significant increase of CD4/CD8 ratio was observed in the late stage of breast tumor models (Figure 1E), we therefore determined the alterations of Foxp3^+^CD4/CD8 T cell ratios in the different tumor developmental stages. As expected, Foxp3^+^CD4/CD8 ratios were dramatically increased with the tumor progression in both tumor models (Figure 2E). These data clearly suggested that the accumulation and increase of CD4^+^ TILs cells in the late breast tumor stages is due to the increases of Th17 and Treg cell populations.

### Distinct distributions of CD4^+^ T cell subsets in peripheral organs in breast tumor-bearing mice

**Figure 3:**
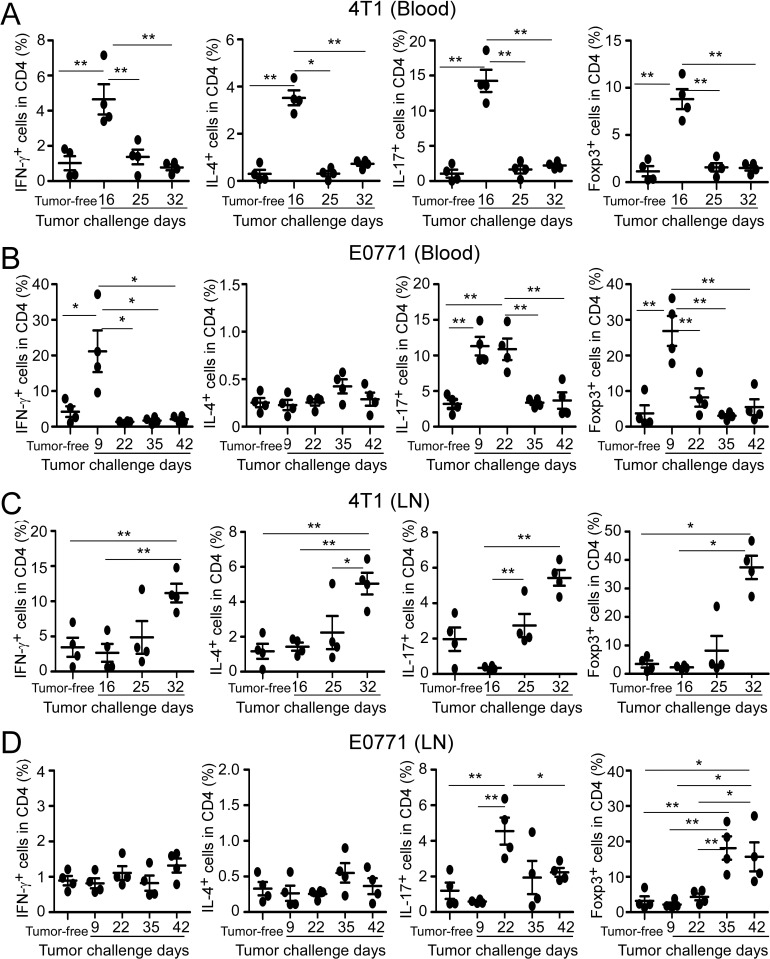
Distributions of CD4^+^ T cell subsets in peripheral blood and draining lymph nodes in breast tumor-bearing mice The dynamic distributions of CD4^+^ T cell subsets in blood (**A**. and **B**.) and draining lymph nodes (**C**. and **D**.) from breast tumor-bearing mice in two models were analyzed at the indicated time points using flow cytometry analyses by gating CD4^+^ population. Tumor-free BALB/c (4T1) and C57BL/6 (E0771) mice were included as controls. The treatment procedure was identical as that described in Figure 1. T cells were isolated from the organs and intracellular staining performed after stimulation with PMA and ionomycin for 5 hours. Results shown in **A.** to **D.** are mean ± SE from four individual mice in each time point. *p < 0.05 and **p < 0.01 between the indicated two groups determined by paired student's t test. Data shown in **A.** to **D.** are representative from three independent experiments with similar results.

We next determined whether CD4^+^ T cell subsets in the other organs could have similar profiles as those in the tumor sites. The dynamic distributions of CD4^+^ T cell subsets in peripheral blood, spleen and draining lymph nodes were analyzed in both normal and tumor-bearing mice. In tumor-free C57BL/6 and BALB/c control mice, there were very limited proportions of CD4^+^ T cell subsets in peripheral blood (Figure 3A and 3B). However, after the tumor challenge, all CD4^+^ T cell subsets in blood had sharp increases at the early stage and then followed to rapidly drop down at the middle or late cancer stages in 4T1 and E0771 tumor-bearing mice, suggesting the hematological changes of CD4^+^ T cell subsets (Figure 3A and 3B). Moreover, compared with tumor-free control, significant increased numbers of white blood cells (WBC) were observed in the peripheral blood of 4T1-bearing mice but not in E0771-bearing mice (Supplemental Figure 3A). In addition to the blood, all four T cell subsets markedly increased in tumor draining lymph nodes at the late stage of 4T1 tumor progression (Figure 3C). Whereas in E0771-bearing mice, only CD4^+^IL17^+^ and CD4^+^Foxp3^+^ subsets had significant increases in tumor draining lymph nodes (Figure 3D). Furthermore, tumor draining lymph nodes of the both breast cancer models harbored high levels of FoxP3^+^Treg subset, which reached the maximal levels at the late cancer stage (Figure 3C and 3D). Notably, all CD4^+^ T cell subsets remained at very low proportions in the spleens in both tumor models, although they had varied distribution levels during tumor development (Supplemental Figure 3B and 3C). These studies clearly suggested that distribution profile of CD4^+^ T subsets in the peripheral organs cannot predict that in the tumor microenvironment.

### Prevalence of both CD4^+^ and CD8^+^ T cells in situ in human breast cancer tissues

**Figure 4:**
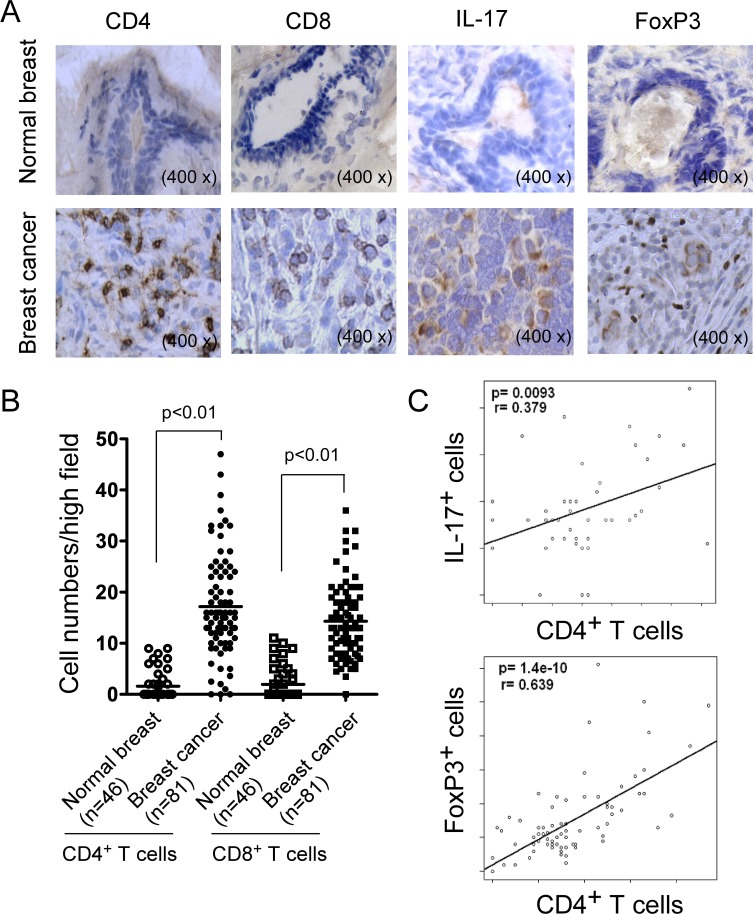
Accumulation of CD4^+^ and CD8^+^ T cells in clinical breast cancer tissues **A.** Representative immunohistochemical staining of CD4^+^, CD8^+^ T cells, as well as IL-17^+^ and FoxP3^+^ cells in normal breast and cancer tissues. Frozen or paraffin-embedded tissue sections were immunohistochemically stained to detect the indicated cells. **B.** Significant increase numbers of CD4^+^ and CD8^+^ T cells in breast cancer tissues (*n* = 81) compared with normal breast tissues (*n* = 46). Numbers of CD4^+^ and CD8^+^ T cells shown are average numbers per high field (magnification 400×) in each tissue sample. The median number of T cells in each group is shown as a horizontal line. Significance was determined by unpaired *t* test. **C.** Scatter diagram analyses showing positive correlations between CD4^+^ T cells and IL-17^+^ and FoxP3^+^ cells in breast cancer tissues. Different types of TILs in frozen sections of breast tumor samples (*n* = 46) were immunohistochemically determined as described in **A.**.

Our findings from mouse breast cancer models suggested that both CD4^+^ and CD8^+^ T cells significantly infiltrated into tumor sites. To further confirm the role of CD4^+^ and CD8^+^ T cells in human breast cancer development and progression, we assessed CD4^+^ and CD8^+^ T cells, as well as IL-17^+^ and Foxp3^+^ cells in breast tumor tissues from cancer patients using the immunohistochemical staining (Figure 4A). In normal breast tissues, very low levels of T cells were detected. In contrast, significantly increased numbers of both CD4^+^ and CD8^+^ T cells were detected in breast tumor tissues, which were consistent with the findings in mouse breast cancer models (Figure 4B). In parallel experiments, we found that very high percentages of IL-17^+^ and FoxP3^+^ cells also existed in breast cancer tissues compared with those in normal breast tissues (Figure 4A and data not shown). Linear correlation analyses further demonstrated that both IL-17^+^ and FoxP3^+^ cells were significantly positively correlated with CD4^+^ TILs (Figure 4C). Notably, relative to IL-17^+^ cells (r = 0.379; P = 0.0093), Foxp3^+^ cells had a more significant correlation with CD4^+^ TILs (r = 0.639; P = 1.4 x10^−10^) in breast cancer patients. These results collectively suggested that both FoxP3^+^ and IL-17^+^ T cells are important components of TILs in breast cancer patients, and that the increase and activation of FoxP3^+^ Treg cells is an important strategy for tumor escaping from anti-tumor immunity during tumor development.

### Associations of CD4^+^ and CD8^+^ T cells with clinicopathologic factors of breast cancer patients

Given that immune system has duel roles of tumor surveillance and promotion during the tumor development [11–14], we investigated clinical significance of CD4^+^ and CD8^+^ T cells in human breast cancer. Clinicopathological factors of breast cancer patients were retrospectively analyzed relative to the levels of the intra-tumoral CD4^+^ and CD8^+^ T cells as well as CD4/CD8 ratios. In addition, cancer-specific survival rates for patients were analyzed in correlation with T cells (CD4^+^, CD8^+^, FoxP3^+^ and IL-17^+^ T cells). As shown in Table 1, intra-tumoral CD4^+^ T cell numbers were positively correlated with advanced tumor stages (p = 2.75 × 10^−5^), large tumor sizes (p = 0.01), positive lymph node status (p = 0.003) and HER2 expression (P = 0.03). In contrast, CD4^+^ T cell infiltration was inversely correlated with RFS (p = 0.07) of breast cancer patients. In addition, we also confirmed that numbers of tumor-infiltrating FoxP3^+^ cells in breast cancer were also positively correlated with tumor stages and lymph nodal status, but strongly negatively correlated with relapse-free survival (RFS) and overall survival (OS) [37]. Interestingly, we did not find any correlation between numbers of tumor-infiltrating IL-17^+^ cells and clinicopathological parameters in breast cancer patients (Data not shown). These results combined with the findings from mouse models suggest that tumor-infiltrating CD4^+^ T cells have dynamic subsets during tumor progression, which may function as a tumor promoter in the late cancer stage.

**Table 1:** Correlations between CD4^+^ and CD8^+^ T cell numbers, CD4/CD8 ratio, and clinicopathologic characteristics in breast cancer patients (n=81)

	CD4			CD8			CD4/CD8		
	≤16	＞16	P	≤13	＞13	P	≤1.2	＞1.2	P
**Age**									
<60 years	23	18		14	25		21	20	
≥60 years	24	15	0.79	21	20	0.25	18	21	0.82
**Tumor stage**									
I	27	3		7	23		24	6	
II	15	15		15	15		10	20	
III	6	15	**2.75×10^−5^**	13	8	**0.02**	6	15	**1.25 ×10^−4^**
**Tumor size**									
<2.1 cm	30	11		15	24		25	16	
≥2.1 cm	17	22	**0.01**	20	21	0.48	14	25	**0.04**
**Nodal status**									
Negative	32	10		11	31		29	13	
positive	16	23	**0.003**	24	15	**2.84×10^−3^**	11	28	**5.58 ×10^−4^**
**ER status**									
Negative	11	12		7	16		9	14	
Positive	37	21	0.28	28	30	0.23	31	27	0.36
**HER2**									
High	13	9		11	11		11	11	
Low	28	15		15	28		24	19	
Negative	2	8	**0.03**	6	4	0.25	2	8	0.12
**Relapse-freeSurvival**									
No-recurrance	38	17		12	35		35	20	
Recurrence	7	10	**0.07**	15	19	**2.67×10^−5^**	2	15	**5.35 ×10^−4^**
**Overall Survival**									
Alive	37	19		17	39		34	22	
Died	11	14	0.11	18	7	**1.14 ×10^−3^**	6	19	**4.92 ×10^−3^**

Note: Evaluated by χ2 test. Abbreviations: ER, estrogen receptor; HER2, human epidermal growth factor receptor 2. Boldface indicates the significance of the p value.

Tumor-infiltrating CD8^+^ T cells have been shown to be an important biomarker for predicting clinical outcome in human breast cancer [32]. As expected, we observed that tumor-infiltrating CD8^+^ T cells were inversely correlated with advanced tumor stages (*p* = 0.02) and positive lymph node status (*p* = 2.84 × 10^−3^), as well as were significantly positively correlated with clinical outcomes of RFS (*p* = 2.67 × 10^−5^) and OS (*p* = 1.14 × 10^−3^) (Table 1). These results further indicated different effects mediated by CD4^+^ and CD8^+^ TILs in anti-tumor immunity and clinical outcomes of breast cancer, and that CD8^+^ is the most critical effector cells. Meanwhile, we also analyzed the CD4/CD8 ratios with those factors and observed that CD4/CD8 ratios were also strongly positively correlated with advanced tumor stages (*p* = 1.25 × 10^−4^), large tumor sizes (*p* = 0.04) and positive lymph node status (*p* = 5.58 × 10^−4^), but negatively correlated with RFS (*p* = 5.35 × 10^−4^) and OS (*p* = 4.92 × 10^−3^) (Table 1), suggesting that CD4/CD8 ratio is an useful and significant prognostic biomarker for assessing clinical outcomes of human breast cancer.

### Opposing roles of CD4^+^ and CD8^+^ T cells in breast cancer progression and outcome

**Figure 5:**
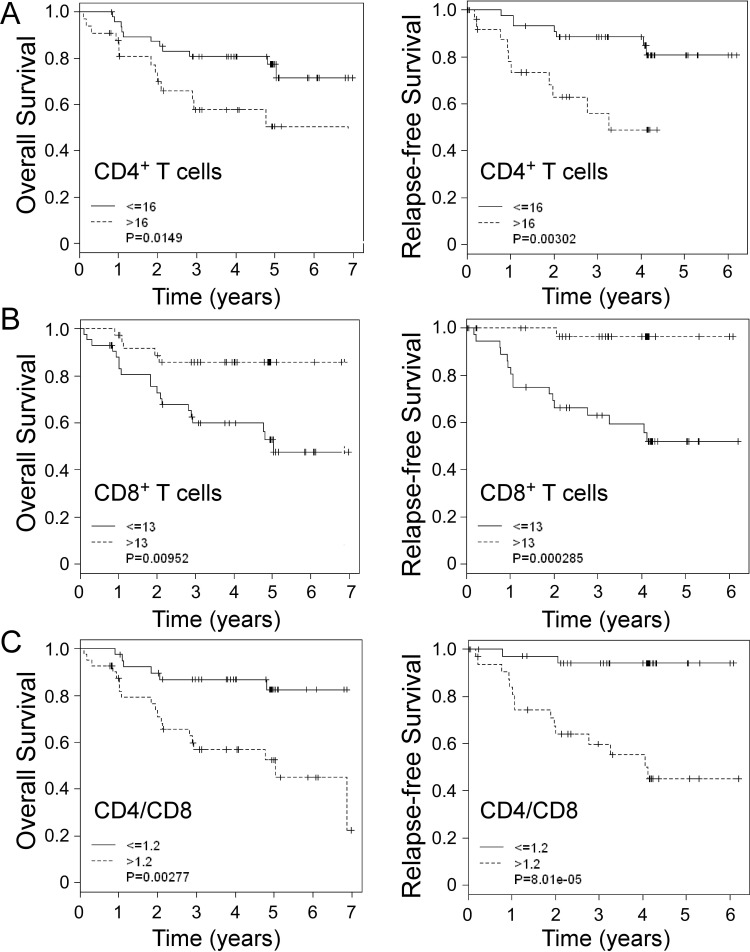
Distinct roles of tumor-infiltrating CD4^+^ and CD8^+^ T cells in predicting clinical outcomes in breast cancer patients Kaplan-Meier analyses of overall survival and relapse-free survival stratified for high and low numbers of tumor-infiltrating CD4^+^ **A.** and CD8^+^ T cells **B.**, as well as CD4/CD8 ratios **C.** in breast cancer patients. The median number of indicated T cells (16 for CD4^+^ T cells, 13 for CD8^+^ T cells and 1.2 for CD4^+^/CD8^+^ ratios) in breast cancer tissues was used as a cutoff point to define high-number and low-number groups. The *p* values were calculated with use of the log-rank test.

To further investigate the possible divergent roles of tumor-infiltrating CD4^+^ and CD8^+^ T cells in breast cancer development, we performed univariate Cox proportional hazard regression analyses of the relationships between CD4^+^ and CD8^+^ T cell levels, other prognostic clinicopathological factors and clinical outcomes in breast cancer patients. We found that lymph node status, tumor stage, tumor-infiltrating CD4^+^ and CD8^+^ T cell numbers, and CD4/CD8 ratio were all significant factors for the prediction of breast cancer outcomes (Supplemental Table 1A). We also performed multivariate Cox regression analyses and confirmed that tumor-infiltrating CD8^+^ T cell levels had independent effects on both OS and RFS (Supplemental Table 1B). In addition, Kaplan-Meier analyses further demonstrated that the 5-year OS and RFS probabilities were both above 80% in CD4^+^ T cells ≤16 (median expression levels in cancer tissue) cancer patients, whereas only about 50% of OS and RFS in CD4^+^ T cells > 16 patients (Figure 5A). In contrast to CD4^+^ T cells, if CD8^+^ T cells were above 13, 5-year OS probability was around 90% and RFS rate was above 95% in cancer patients, whereas only 45% of OS and 50% of RFS in CD8^+^ T cells ≤13 cancer patients (Figure 5B). Furthermore, we obtained that breast cancer patients with high levels of CD4/CD8 ratio ( > 1.2) had significantly poor cancer-specific OS and RFS probabilities, which was very similar as those in CD4^+^ T cells (Figure 5C). We also determined whether CD4^+^ and CD8^+^ T cells had different roles and prognostic value for clinical outcomes in breast cancer patients with different ER or HER2 expression status. However, Kaplan-Meier analysis results showed that CD4^+^ and CD8^+^ T cells as well as CD4/CD8 ratios in ER^+^ and HER2^+^ patients had very similar patterns for the predictions of clinical outcomes as those in the total breast cancer patients, suggesting that roles of both CD4^+^ and CD8^+^ T cells in breast cancer pathogenesis and progression are independent of ER and HER2 expression (Data not shown). Taken together, our results clearly indicate that CD4^+^ and CD8^+^ T cells have distinctly different roles in controlling breast cancer progression and outcomes.

**Figure 6:**
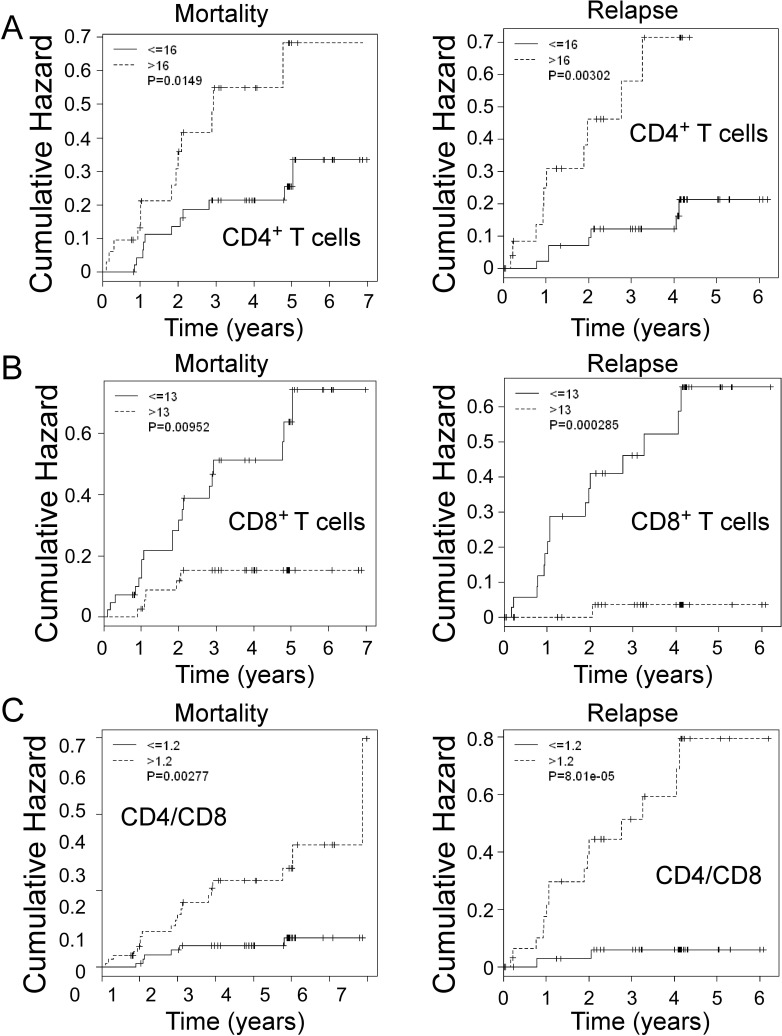
Prognostic values of CD4^+^ and CD8^+^ T cells, as well as CD4/CD8 ratio for the risks of breast cancer mortality and relapse in all breast cancer patients during the follow-up period An increasing annual HR for mortality and relapse per year in the breast cancer patients (*n* = 81) who have high numbers of tumor-infiltrating CD4^+^ T cells **A.**, low numbers of tumor-infiltrating CD8^+^ T cells **B.** or high CD4/CD8 ratios **C.** throughout the entire follow-up period. The *p* values were calculated with use of the log-rank *test*.

In addition to retrospectively analyzing the associations with OS and RFS in breast cancer patients, we further determined the prognostic value of intra-tumoral CD4^+^ and CD8^+^ T cells for the prediction of risk of breast cancer development during the follow-up period. As shown in Figure 6A, there were significantly increased cumulative hazard ratios with the increasing follow-up years for mortality and relapse in the cancer patients containing high levels of tumor-infiltrating CD4^+^ T cells (CD4^+^ T cells > 16). In contrast, cancer patients containing low levels of tumor-infiltrating CD4^+^ T cells (≤16) had decreased mortality ( < 30%) and relapse ( < 20%) throughout the entire 5 year follow-up. Furthermore, the level of tumor-infiltrating CD8^+^ T cells was also an important prognostic factor for the prediction of breast cancer development. Cancer patients containing high levels of tumor-infiltrating CD8^+^ cells ( > 13) had significantly low mortality and relapse with cumulative hazards of 0.1 and 0.05, respectively throughout the entire 5 year follow-up, whereas patients containing low levels of CD8^+^ cells (≤13) may die or relapse with a cumulative hazard of 0.7 (Figure 6B). Notably, intra-tumoral CD4/CD8 ratio is still a significant and valuable clinical biomarker for identifying the risk for late-relapse and survival of breast cancer patients. Cancer patients containing low levels of CD4/CD8 ratio (≤1.2) had significant very low mortality and relapse with cumulative hazards below 0.05 throughout the entire 5 year follow-up (Figure 6C). These results further suggest that intra-tumoral CD4^+^ and CD8^+^ T cells have opposing prognostic roles in controlling breast cancer development.

## DISCUSSION

Dissecting the dynamics and functional roles of different subsets of TILs in the tumor suppressive microenvironment should facilitate our better understanding of immunopathogenesis of cancer and the development of effective strategies for anti-tumor immunotherapy. Although the “cancer immunoediting” concept has precisely described how host immune system dynamically interacts with tumor cells and its dual roles on tumor development and progression in general, identifying the replicability of this principal in individual cancer type is critical for the effective clinical interventions [11–14]. In the current study, we investigated CD4^+^ T cell subsets and CD8^+^ T cells in tumor sites and other multiple organs during the course of tumor development and progression in two breast cancer models. Our results suggested that both CD4^+^ and CD8^+^ T cells are dynamically involved in the immune responses in breast cancer. In the early stage of breast tumor development, Th1 and CD8^+^ T cells were dominant populations in TILs which may perform immunosurveillance. However, in the late cancer stage, CD4^+^ TILs significantly increased in numbers and its dominant subsets changed to Treg and Th17 cells, which may contribute to tumor promotion. Moreover, our results from breast cancer patients confirmed that CD8^+^ T cells are the key effector cells for anti-tumor immunity and positively associated with better clinical outcomes. Furthermore, CD4^+^ and CD8^+^ T cells have opposing prognostic effects for breast cancer patients. These studies collectively suggest the dynamics and distinct roles of CD4^+^ and CD8^+^ T cells in directing breast cancer progression and outcomes.

Identification of suitable animal models mimicking clinical cancers is critical for understanding the cancer biological behaviors and cell interactions within tumor microenvironments, as well as for facilitating the studies of preclinical assessment of cancer drug discovery and novel immune therapeutic strategies on human cancers. In this study, we utilized two distinct murine mammary cancer cell lines 4T1 and E0771 with different pathologic types to establish breast cancer models and investigate the dynamics of immune cells during tumor development. 4T1 cells originally isolated from BALB/c mice share many characteristics with naturally occurring human breast cancer and metastasize to distant organs via the haematogenous route, making it a good model for mimicking the metastatic and advanced stages of human breast cancer [33, 38]. E0771 cell line is a medullary breast adenocarcinoma cell type, and syngeneic to C57BL/6 mice, representing the human medullary breast cancer with spontaneous development [34]. In our efforts to understand the interactions and changes of T cells with breast tumor progression, we found that there were several differences of immune responses in peripheral organs within these two breast cancer models. We observed significant increased numbers of white blood cells in the peripheral blood of 4T1-bearing mice but not in E0771-bearing mice during the tumor progression (Supplemental Figure 3A). In addition, all CD4^+^ T cell subsets markedly increased in tumor draining lymph nodes at the late stage of 4T1 tumor progression, whereas in E0771-bearing mice, only CD4^+^IL17^+^ and CD4^+^Foxp3^+^ subsets had significant increases in tumor draining lymph nodes (Figure 3C and 3D). However, the overall changes and dynamic distributions of tumor-infiltrating CD4^+^ and CD8^+^ T cells had similar trends in both breast cancer models except the earlier peak of Th1 cells in the E0771 model than that in the 4T1 tumor model with tumor progression. Our studies suggest that both of these breast tumor models are useful for the studies focusing on the interactions between immune system and tumor cells in the tumor microenvironment.

Improved understanding of the role of CD4^+^ T cells in anti-tumor immunity is challenging in tumor immunology research and the results have been controversial. Besides the traditional functions of Th1 and Th2 cells in priming and helping tumor-specific CD8^+^ T cells and B cells, recent discovery of Th17 and Treg cells has not only resulted in an explosion of cancer immunological research but also markedly changed our conventional thinking of the role of CD4^+^ T cells in the pathogenesis of cancer development [17–25]. In the current study, we provided critical information regarding CD4^+^ T cells in the immunoeditting process during breast cancer development and progression both in animal models and in cancer patients. We first showed that numbers of CD4^+^ T cells are significantly increased with breast cancer development, suggesting an active involvement of tumor-induced immune responses. Importantly, besides the increase in numbers, the cell subsets of CD4^+^ T cells also dynamically changed, indicating distinct functions of CD4^+^ T cells in the different tumor developing stages. In early tumor stages, Th1 cells are the dominant population of CD4^+^ T cells, perhaps important for immunosurveillance; while in the advanced tumor stages, FoxP3^+^ Treg and Th17 cells become the dominant populations. It is well recognized that tumor-infiltrating Treg cells are a major obstacle for the success of tumor immunity and immunotherapy [21–25]. Therefore, our studies suggested that in the late tumor stage, CD4^+^ T cells may become more important for promoting tumor growth. This assertion was further confirmed in our retrospective studies in breast cancer patients, demonstrating that intra-tumoral CD4^+^ T cell numbers were positively correlated with advanced tumor stage, large tumor sizes, and positive tumor metastasis, but were inversely correlated with survival of breast cancer patients. In addition, we also confirmed that numbers of tumor-infiltrating FoxP3^+^ cells in breast cancer were also positively correlated with tumor stages and lymph nodal status, but negatively correlated with RFS and OS [37]. Notably, although we demonstrated that Th17 cells are a dominant population in CD4^+^ TILs in both breast cancer models, we did not find any correlations between numbers of tumor-infiltrating IL-17^+^ cells with clinicopathological parameters in breast cancer patients. This may due to the small sample size in our current study. In addition, we observed that CD4^+^ T cell subsets have distinct distributions in peripheral organs (blood, spleen and lymph nodes), as in tumor sites, further indicating that immune profiles in peripheral organs cannot predict immune subsets *in situ* within the tumor microenvironment. Taken together, our studies suggest that CD4^+^ T cells have dynamic roles and subset distributions during breast cancer development and progression. Furthermore, dissecting the cell subsets and densities of TILs *in situ* in the tumor microenvironment should be more important and valuable for the prediction of clinical outcomes and the development of novel immunologic targeting therapies.

Our current studies further confirmed that CD8^+^ T cells are the key effector cell population controlling effective anti-tumor immunity and patient clinical outcomes. Our studies from breast cancer mouse models and cancer patients clearly demonstrated significant accumulation of CD8^+^ T cells in the tumor sites. We observed that tumor-infiltrating CD8^+^ T cells were negatively correlated with advanced tumor stages and positive tumor metastasis status, but significantly positively correlated with clinical outcomes of RFS and OS. In support of our conclusions, studies from other groups have shown that high amounts of tumor-infiltrating CD8^+^ T cells have a favorable prognosis in breast, ovarian and colorectal cancers [27, 32, 39, 40]. Our results also suggest that tumor-infiltrating CD4^+^ and CD8^+^ T cells have distinct roles for control of tumor progression and clinical outcomes. Besides the retrospective analyses of breast cancer patients, the results from the two breast cancer murine models showed that the proportion of CD8^+^ T cells was more dominant than CD4^+^ T cells in TILs in the early stages of tumor development. However, CD4^+^ T cells more rapidly infiltrated into the tumor sites than CD8^+^ T cells with the tumor progression, and therefore became the more dominant population in late stages of tumor development (Figure 1D). Additional evidence supporting different roles of CD4^+^ and CD8^+^ T cells in the pathogenesis of breast cancer includes the dynamics of CD4/CD8 proportions with progression of tumor development. We observed markedly increased CD4/CD8 ratios at late tumor stages compared with those in the early tumor stages in both 4T1 and E0771 breast tumor models. Furthermore, results from clinical cancer patients showed that CD4/CD8 ratios were strongly positively correlated with the advanced tumor stage, large tumor sizes and positive lymph node status, but negatively correlated with RFS and OS. Based on these results, the major challenge for augmenting the success of anti-tumor immunity and immunotherapy against breast cancer is to develop effective strategies to increase CD8^+^ T cells and keep the optimal balance of CD4/CD8 in the tumor microenvironment. Notably, our current studies also indicate that intra-tumoral CD4^+^ and CD8^+^ T cells, as well as the ratio of CD4/CD8 in TILs are all valuable and significant prognostic biomarkers for predicting the outcomes of human breast cancer.

## MATERIALS AND METHODS

### Cell lines

Breast tumor cell lines 4T1 and E0771 were obtained from the American Tissue Culture Collection (ATCC) and maintained in RPMI 1640 medium containing 10% fetal calf serum (FCS) and penicillin-streptomycin (Invitrogen, Inc. San Diego, CA).

### *In vivo* studies

BALB/c and C57BL/6 mice (6 to 8-wk-old female) were purchased from The Jackson Laboratory and maintained in the institutional animal facility. All animal studies have been approved by the Institutional Animal Care Committee. Mouse breast tumor 4T1 (1 × 10^5^) or E0771 (2 × 10^5^) cells in 100 μl of buffered saline were subcutaneously injected into in the mammary fat pad of BALB/c (4T1) and C57BL/6 mice (E0771), respectively. Five to ten mice were included in each group. Tumor volumes were measured every 3 days. When the tumor volumes reached the indicated sizes in diameters (4T1: 3-5 mm, 12-15 mm and 18-20 mm; E0771: 3-5 mm, 8-10 mm, 12-15 mm and 18-20 mm), the tumor-bearing mice were sacrificed. Blood, lymph nodes (LN), spleens (SP) and tumor tissues were harvested and mononuclear cells were purified for subsequent phenotypic and cytokine profile analyses *in vitro*, as previously described [25, 41–43]. In addition, lymphocytes from different organs of normal littermates were also harvested and used as negative controls.

### Flow cytometry analysis

The expression markers on T cells were determined by FACS analyses after surface or intracellular staining with anti-mouse specific antibodies conjugated with Alexa Fluor 488, PE, FITC, allophycocyanin (APC), or PerCP.cy5.5. These mouse antibodies included: anti-CD3, anti-CD4, anti-CD8, anti-IFN-γ, anti-IL-4, anti-IL-17, and anti-FoxP3, which were purchased from BD Biosciences. For intracellular staining, T cells were stimulated with PMA (1 μg/ml) and ionomycin (2 μg/ml) (Sigma-Aldrich) for 5 hours in the presence of GolgiStop (BD Biosciences) before the intracellular staining with antibodies. All stained cells were analyzed on a LSR II cytometer (BD Bioscience) and data analyzed with FlowJo software (Tree Star).

### Patients and sample collection

Tumor samples were obtained from breast cancer patients treated at Saint Louis University Department of Surgery from 2004 to 2012 who have given informed consents for enrollment in a prospective tumor procurement protocol approved by the Saint Louis University Institutional Review Board. Total of 81 tumor tissues from different stages of identified primary breast cancer were collected for this study. Whenever feasible without interfering with histopathologic analysis for ongoing clinical decision making, paired fresh tumor tissues and normal breast tissues were obtained perioperatively and snapped frozen in liquid nitogen (*N* = 46). For patients from whom fresh tissues were not obtained, paraffin blocks of tumor tissues were obtained for analysis (*N* = 35). Patient clinical data were also collected for analysis.

### Immunohistochemical staining and quantification method

The cell populations of CD4^+^ and CD8^+^ T cells, IL-17^+^ and FoxP3^+^ cells in cancer and normal tissues (frozen or paraffin-embedded sections) were determined using immunohistochemical staining with the Histostain®-Plus 3rd Gen IHC Detection Kit (Invitrogen, CA), as we described previously [37, 42, 44]. For frozen section staining, the following monoclonal antibodies were used: mouse anti-human IL-17 (clone 1C12), FoxP3 (clone 236A/E7), CD4 (clone RPA-T4), and CD8 (clone RPA-T8) (eBioscience, San Diego, CA) monoclonal antibodies, at diluted concentrations of 1: 50, 1: 50, 1:100 and 1:100, respectively. For paraffin-embedded tumor sections, the following monoclonal antibodies were used: FoxP3 (clone 236A/E7), CD4 (clone BC/1F6), and CD8 (clone 144B) (Abcam, Boston, MA) monoclonal antibodies at a diluted concentration of 1: 50. Controls were performed by incubating slides with the isotype control antibody instead of primary antibodies, or second antibody alone. Normal breast tissues served as controls. Expressions of CD4^+^, CD8^+^ T cells, and IL-17^+^ and FoxP3^+^ cells in tissues were evaluated manually using a computerized image system composed of a Leica ICC50 camera system equipped on a Leica DM750 microscope (North Central Instruments, Minneapolis, MN). Twenty fields (400 ×, magnification) of each tumor tissue section, including both cancer nest and stroma areas were counted and summed, and the means of positive cell numbers per field reported. The counting was performed by three independent investigators (C. Ma, J. Ye and F. Wang) who had no previous knowledge of the patient clinical backgrounds, and the results were averaged.

### Statistical analysis

For *in vivo* animal experiments, data are expressed as mean ± standard deviation (SD). The significance of difference between groups was determined by paired or unpaired two-tailed Student's *t*-test or the one-way analysis of variance (ANOVA). Differences were considered significant for p values less than 0.05. For breast cancer tissue analyses, the median expression of each TIL population (16 for CD4^+^ T cells, 12 for FoxP3^+^ cells, 8 for IL-17^+^ cells, and 13 for CD8^+^ T cells) in breast cancer tissues was used as a cutoff point to define the TIL-high and TIL-low groups, as we previously described [37]. Pearson's Chi-square test was used to prospectively analyze the correlations between the cell number of each TIL and clinical features, including age, nodal status, tumor size, tumor stage, estrogen receptor (ER) status, epidermal growth factor receptor 2 (HER2) positivity, relapse-free survival (RFS) and overall survival (OS). OS was determined from the date of surgery to the date of death by any cause or to the date of the last follow-up. RFS was measured as the length of time from surgery to the date of relapse. For all categorical predictors (including the cell numbers dichotomized by medians), the log-rank test was used to perform univariate survival association analyses for OS and RFS. Survival and relapse-free probability and cumulative hazard associated with prognostic factors for OS and RFS were estimated by the Kaplan-Meier method, and hazard ratios were estimated by a Cox proportional hazard regression model. Data processing and statistical analyses were performed using SAS 9.1 and R 2.13.0. Statistical significance was defined when alpha < 0.05 (2-tailed).

## SUPPLEEMENTARY MATERIAL FIGURES AND TABLE


