# Supplementary material for: CD4+ and CD8+ T cells have opposing roles in breast cancer progression and outcome
Source: Oncotarget. 2015 Apr 29;6(19):17462–78. doi: 10.18632/oncotarget.3958 (PMC4627321; doi:10.18632/oncotarget.3958)
Supplement: Supplementary file 1 [file oncotarget-06-17462-s001.pdf]

# CD4+ and CD8+ T cells have opposing roles in breast cancer progression and outcome

**Supplementary Material**

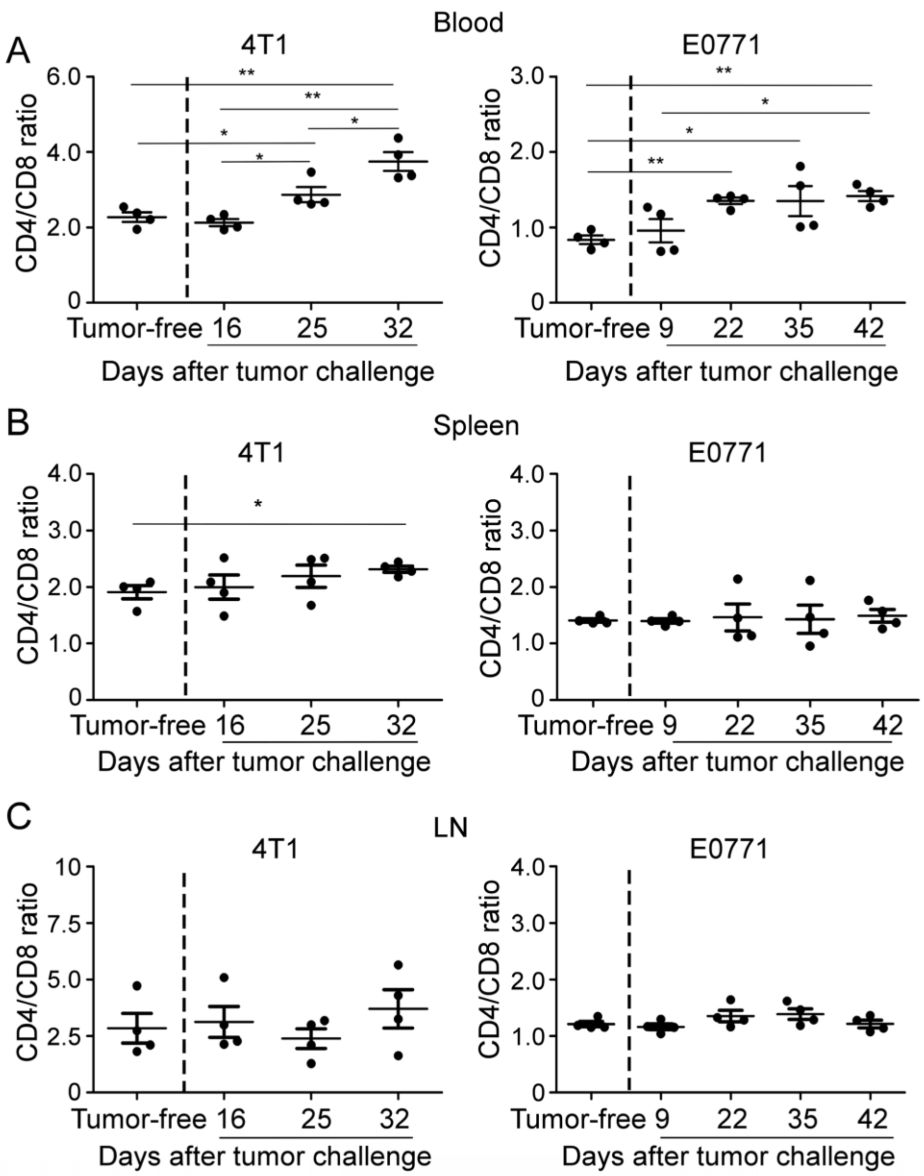


**Supplemental Figure 1**

**(A) to (C) Dynamic changes of CD4+ to CD8+ T cell ratios in peripheral blood (A), spleen (B) and draining lymph nodes (C) at the different stages of tumor development in two models.** Tumor- free BALB/c (4T1) and C57BL/6 (E0771) mice were included as controls. The treatment procedure was identical as that described in Figure 1. T cells were isolated from the organs and proportions of CD4+ and

$CD8^+$ T cells detected at the indicated time points using flow cytometry analyses by gating $CD3^+$ population. Results shown in (A) to (C) are mean ± SE from four individual mice in each time point. * $p < 0.05$ and ** $p < 0.01$ between the indicated two groups determined by paired student's T-test.

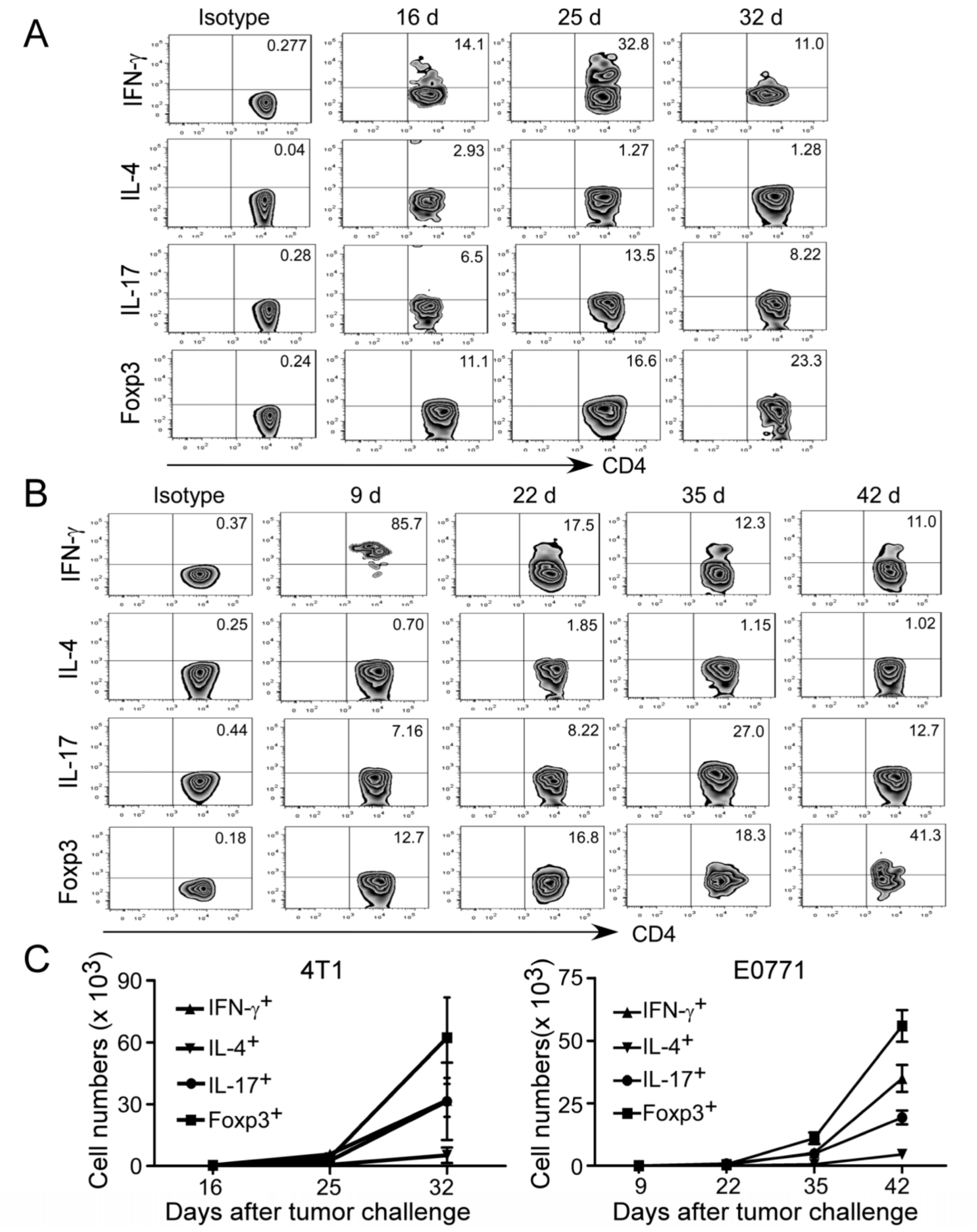


**Supplemental Figure 2**

**Dynamic analyses of tumor-infiltrating CD4$^+$ T cell subsets in mouse breast cancer models.**

**(A)** and **(B)** Proportions of CD4$^+$ T cell subsets in TILs were analyzed at the indicated time points using flow cytometry analyses by gating CD4$^+$ population. Representative FACS graphs from 4T1- bearing mice (A) and E0771-bearing mice (B) are shown. The treatment procedure was identical as that described in

Figure 1. TILs were isolated and intracellular staining performed after stimulation with PMA and ionomycin for 5 hours. **(C)** Total cell numbers of $CD4^+$ T cell subsets were concluded. Data shown are mean ± SE from four individual mice in each group.

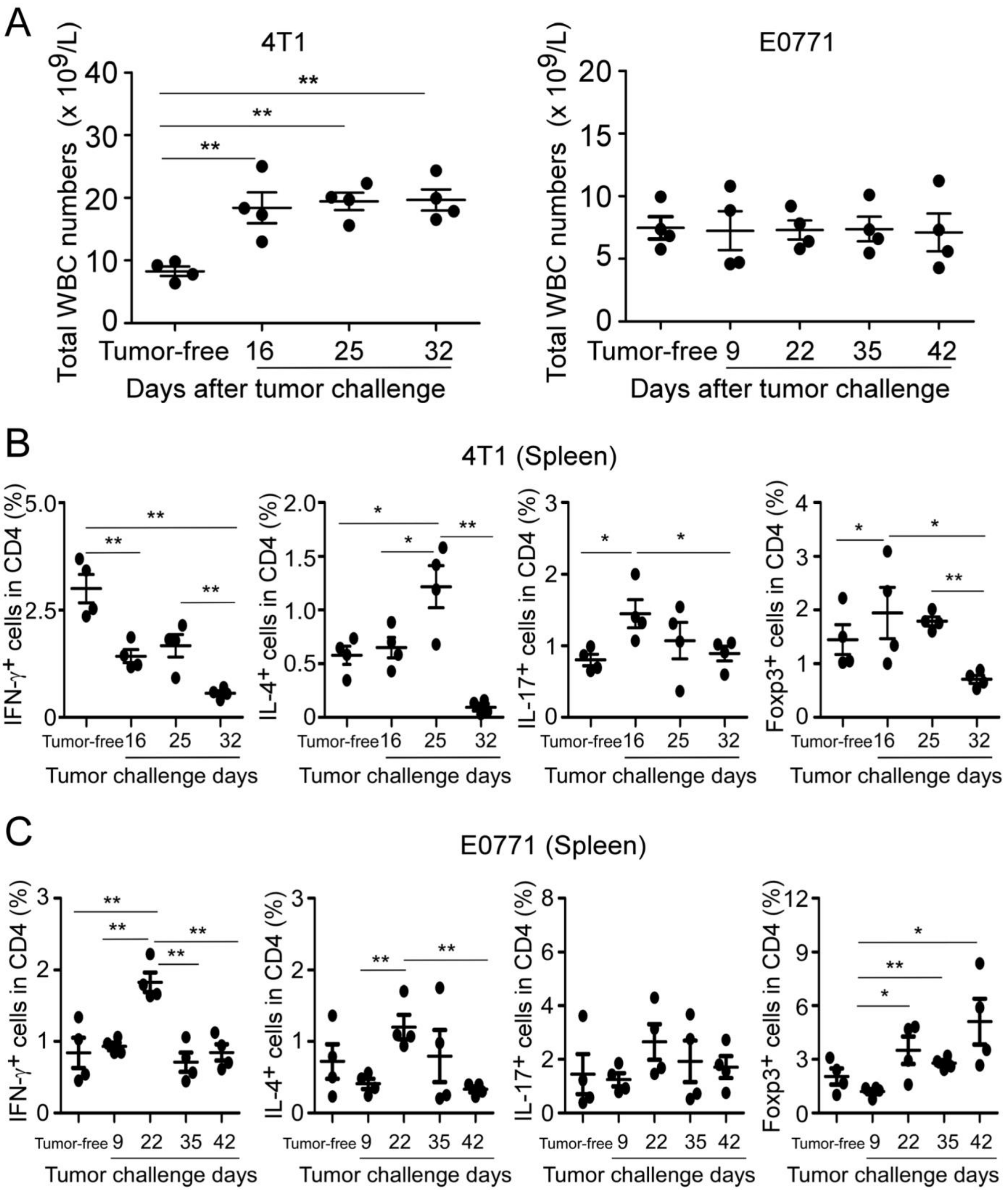


**Supplemental Figure 3**

**Distributions of CD4$^+$ T cell subsets in blood and spleens in breast tumor-bearing mice.**

**(A)** Peripheral blood from two mouse breast tumor models were collected and total white blood cell numbers counted. The mouse treatment procedure was identical as that described in Figure 1. **(B)** and **(C)** The dynamic distributions of $CD4^+$ T cell subsets in spleens from breast tumor-bearing mice in two models were analyzed at the indicated time points using flow cytometry analyses by gating $CD4^+$ population. The treatment procedure was identical as that described in Figure 1. T cells were isolated from the organs and intracellular staining performed after stimulation with PMA and ionomycin for 6 hours. Results shown in (A) to (C) are mean ± SE from four individual mice in each time point. Tumor-free BALB/c (4T1) and C57BL/6 (E0771) mice were included as controls. * $p < 0.05$ and ** $p < 0.01$ between the indicated two groups determined by paired student's T test.

**Supplemental Table 1.**

**A. Univariate analyses of factors associated with Relapse-free Survival and Overall Survival in breast cancer patients (n=81).**

| | Relapse-free Survival | | | Overall survival | | |
|---|---|---|---|---|---|---|
| **Variables** | **HR** | **95% CI** | **p** | **HR** | **95% CI** | **p** |
| **CD4 (>16)** | 3.92 | 1.48 to 10.36 | **0.005** | 2.61 | 1.17 to 5.82 | **0.02** |
| **CD8 (>13 )** | 0.06 | 0.01 to 0.47 | **6.71E-05** | 0.32 | 0.13 to 0.79 | **0.007** |
| **CD4/CD8 (>1.2 )** | 10.87 | 2.46 to 47.23 | **5.01E-05** | 3.73 | 1.48 to 9.41 | **0.002** |
| **Stage ( III *vs* I + II)** | 11.94 | 4.31 to 33.07 | **1.53E-06** | 6.72 | 2.88 to 15.71 | **1.65E-05** |
| **Nodal (Positive )** | 16.71 | 3.76 to 74.26 | **1.35E-06** | 6.97 | 2.58 to 18.79 | **1.15E-05** |

Note: Results obtained using the Cox proportional hazard regression model. Boldface indicates the significance of the *p* value.

**B. Mulvariate analyses of Hazard Ratios with Relapse-free Survival and Overall Survival in breast cancer patients (n=81).**

| | Relapse-free Survival | | | Overall survival | | |
|---|---|---|---|---|---|---|
| **Variables** | **HR** | **95% CI** | **p** | **HR** | **95% CI** | **p** |
| **CD4 (>16)** | 1.37 | 0.35 to 5.25 | 0.65 | 1.37 | 0.49 to 3.81 | 0.55 |
| **CD8 (>13 )** | 0.05 | 0.01 to 0.51 | **0.01** | 0.28 | 0.09 to 0.93 | **0.04** |
| **CD4/CD8 (>1.2)** | 0.65 | 0.08 to 5.48 | 0.68 | 0.69 | 0.17 to 2.78 | 0.60 |
| **Stage ( III *vs* I + II)** | 4.54 | 1.04 to 22.02 | **0.04** | 2.76 | 0.99 to 7.71 | **0.05** |
| **Nodal (Positive)** | 9.46 | 1.19 to 74.74 | **0.03** | 4.61 | 1.29 to 16.44 | **0.02** |

Note: Results obtained using the Cox proportional hazard regression model. Boldface indicates the significance of the *p* value.
